# The Long Noncoding RNA LOXL1-AS1 Promotes the Proliferation, Migration, and Invasion in Hepatocellular Carcinoma

**DOI:** 10.1155/2020/4182092

**Published:** 2020-12-17

**Authors:** Jiang Liu, Chengtong Zhai, Degan Liu, Jianhua Liu

**Affiliations:** The Affiliated Xinghua People's Hospital, Medical School of Yangzhou University, Yangzhou, Jiangsu, China

## Abstract

**Objective:**

To investigate the expression of long noncoding RNA lysyl oxidase-like 1-antisense 1 (LOXL1-AS1) in hepatocellular carcinoma tissues and its effect on cell proliferation, migration, and invasion.

**Methods:**

Quantitative real-time PCR was used to analyze the expression of LOXL1-AS1 RNA in tumor tissues, adjacent normal tissues, and cell lines. MTT assay, colony formation assay, flow cytometry analysis, transwell assays, and lentivirus-mediated RNA interference (RNAi) technology were used to evaluate cell proliferation and migration.

**Results:**

In the present study, we observed that the expression level of LOXL1-AS1 in hepatocellular carcinoma tissue was significantly higher than that in adjacent nontumor tissues, and its expression in three hepatic carcinoma cell lines was obviously higher than that in a normal cell line. In addition, in the Hep-G2 cell line, LOXL1-AS1 downregulation significantly inhibited cell proliferation in the light of the MTT and colony formation assays in vitro, which was consistent with animal experiment in vivo. What is more, cell migration was also inhibited in vitro in Matrigel Transwell Assay by LOXL1-AS1 knockdown, which might be partly attributed to the reduction of MMP-2 and MMP-9 protein expressions. Finally, cell cycle analysis revealed that knockdown of LOXL1-AS1 induced significantly a G0/G1 phase cell cycle arrest, which might be partly attributed to the downregulation of Cdc2, Cdc25A, and cyclin B1 protein expression.

**Conclusion:**

In conclusion, we demonstrated that reduced LOXL1-AS1 expression could inhibit hepatocellular carcinoma cell proliferation, migration, and invasion. The application of RNAi targeting LOXL1-AS1 might be a potential treatment strategy in advanced cases.

## 1. Introduction

Hepatocellular carcinoma (HCC) is one of the most common malignancies that seriously threaten the human health. It is reported that it is the third leading cause of cancer-related mortality, and its incidence has an ascending trend [1]. HCC is a highly angiogenic malignant tumor associated with aberrant angiogenesis, cell cycle dysregulation, and evasion of apoptosis. Despite tremendous advances in diagnosis and treatment [2], the complex molecular pathogenesis of HCC remains poorly understood mainly on account of multiple genetic and epigenetic alterations, gene mutations, chromosomal aberrations, and aberrant molecular pathways [3]. More and more evidence have demonstrated that lncRNAs are reckoned as crucial determinants of HCC occurrence and development.

Recently, lncRNAs have been increasingly recognized to be a kind of novel noncoding RNA molecules which are longer than 200 nucleotides in length, playing an important regulatory role in gene expression and tumorigenesis in various tumor types, including lung cancer, hepatocellular carcinoma, and colorectal cancer [4–11]. Another research revealed that lncRNAs might regulate significant biological processes embracing cell proliferation, differentiation, survival, and apoptosis in both *trans* and *cis* [12]. lncRNAs appeared to be associated with tumor suppressive and oncogenic functions in diverse cancer types [13]. Two studies have indicated that lncRNAs could regulate biological processes of HCC [14, 15]. Another group implied that some lncRNAs might be used as novel potential biomarkers for HCC diagnosis and prognosis as well as predictors of curative efficacy evaluation [16]. However, taking extensive existence into consideration, the expression and role of lncRNAs in HCC remain unclear and to be further investigated.

Up to date, most researches about long noncoding RNA LOXL1 have focused on exfoliation syndrome or glaucoma. LOXL1-AS1 is a genomic sequence encoding the opposite strand of LOXL1. One research finding revealed that LOXL1-AS1 played a functional role in cellular stress response [17]. However, little is known about the role of LOXL1-AS1 in tumor formation and development. In the present study, we demonstrated that reduced LOXL1-AS1 expression could inhibit cell proliferation and migration and identified it as a poor prognostic biomarker in hepatoma. The application of RNAi targeting LOXL1-AS1 might be a potential treatment strategy in advanced cases.

## 2. Materials and Methods

### 2.1. Clinical Specimens

15 cases of hepatocarcinoma tissues and corresponding adjacent tumor samples were obtained from patients who received surgical resection from 2015 to 2018 at Affiliated Xinghua People's Hospital of Yangzhou University. Before surgical resection, patients did not receive radiotherapy and chemotherapy. Consent had been obtained from all patients before this study. All tissues were snap frozen and stored at −80°C refrigerator. This study was approved by the Ethics Committee of Nanjing Medical University. The clinicopathologic features of patients were summarized in Table 1.

### 2.2. Cell Culture

Hepatocarcinoma cell lines Hep-G2, SMMC7721, and SK-HEP1 as well as HEK-293 cell lines were purchased from ATCC of China. Hep-G2, SMMC7721, and HEK-293 cells were cultured in DMEM which included 12%-15% heat-inactivated FBS (*v*/*v*), 100 U/ml penicillin, and100 *μ*g/ml streptomycin. SK-HEP1 cells were grown in DMEM supplemented with 10% fetal bovine serum, 100 *μ*g/ml streptomycin and 100 units/ml penicillin. All cells were cultured in a humidified incubator containing 5% CO_2_ at 37°C.

### 2.3. Lentiviral Vector Construction and Infection

The short hairpin RNA (shRNA) sequence targeting human LOXL1-AS1 was 5′-gtggacaaatcataactgaa-3′ and control sequence was 5′- TTCTCCGAACGTGTCACGT -3′, which were no significantly similar to human gene sequences. A shRNA was synthesized and inserted into pFH1UGW lentivirus vector including a H1 promoter upstream of restriction sites (NheI/PacI) and a CMV-driven EGFP reporter gene.

Recombinant lentiviruses transfected with sh-LOXL1-AS1 or sh-control (sh-LOXL1-AS1 or NC) were from the Genechem Company. After transfection 3 days, the viruses were concentrated by centrifugation and used for subsequent studies.

### 2.4. Quantitative Real-Time PCR (qRT-PCR)

Total RNA was extracted from the tissue samples and cultured cells with TRIzol reagent (Invitrogen).We used random primers and the M-MLV Reverse Transcriptase (Invitrogen) to synthesize first-strand cDNA from 2 *μ*g of total RNA. Primers for detection of LOXL1-AS1 and *β*-actin (internal control) are as follows: LOXL1-AS1 (sense): 5′-AGATGGCTCCTGAGAGTGCT-3′, LOXL1-AS1 (antisense): 5′-TTCTGGGACCCCCTCCTATC-3′; *β*-actin (sense): 5′-GTGGACATCCGCAAAGAC -3′, *β*-actin (antisense): 5′- AAAGGGTGTAACGCAACTA -3′. RNA expression was measured by qRT-PCR using the SYBR Green method (Takara). The results were normalized to the expression of *β*-actin.

### 2.5. MTT Assay

After 5 days of cell culture, cells in all three groups were trypsinized and resuspended for 3-(4,5-dimethylthiazol-2-yl)-2,5-diphenyltetrazolium bromide (MTT) assay. To be brief, 100 *μ*l of cells were seeded into a 96-well plate with a concentration of 2,000 cells per well and incubated at 37°C for 5 days. The number of viable cells was detected every day. At each time point, 10 *μ*l of 5 mg/ml MTT was added into each well, and incubation was continued for 3 h. Then, the medium was removed carefully; 100 *μ*l of acidified isopropanol (in 0.01 M HCl) was added into each well at the end of incubation to dissolve the formazan crystals. The absorbance was detected at 595 nm on the spectrophotometer.

### 2.6. Colony Formation Assay

Both transfected and nontransfected Hep-G2 cells (400 cells/well) were seeded into 6-well plates. The cells were cultured for about 14 days, fixed with 4% paraformaldehyde, and stained with 0.1% crystal violet. After washing, the plates were air dried. The stained colonies were photographed with a microscope (Leica). The total number of colonies (>50 cells/colony) was counted. The experiments were performed in triplicate.

### 2.7. Transwell Migration Assay

Cell migration ability was assessed using 6.5 mm transwell chambers with a pore size of 8 *μ*m. The assays were performed according to the manual instructions. In short, 2 × 10^4^ Hep-G2 cells from each group were suspended without serum medium and then placed into the upper chamber. The lower chamber was brimmed with medium containing 10% FBS. After incubation for 48 hours, the migrated cells in the lower chamber were fixed in 4% paraformaldehyde, stained via 0.1 mg/ml crystal violet liquor, and counted under a microscope (20x objective lens). Five random visual fields were counted for each well, and then, the average was determined. The experiments were performed at least three times.

### 2.8. Cell Cycle Analysis

Both transfected and nontransfected Hep-G2 cells were trypsinized, then washed twice with PBS, and fixed in 70% ethanol at 4°C overnight. After fixation, the cells were washed and resuspended in cold PBS and then incubated in a solution of 1 mg/ml propidiumiodide and 10 mg/ml RNase (Sigma-Aldrich) for 30 min at 37°C in the dark. Finally, the DNA content was detected by flow cytometry. The percentage of cells in the G0/G1, S, and G2/M phases was determined by Cell Quest acquisition software.

### 2.9. Western Blot Analysis

Cellular proteins were extracted from Hep-G2 cells (Con, NC, and sh-LOXL1-AS1) in lysis buffer (Beyotime). The cell proteins were separated by SDS-PAGE and transferred to poyvinylidene difluoride membranes (Millipore). The membranes were blocked and then probed with primary antibodies at 4°C overnight. The following primary antibodies were used in this study: anti- CyclinB1, anti-Cdc25A, anti-Cdc2, anti-MMP-2, anti-MMP-9, and anti-*β*-actin (all from Bioworld Technology). *β*-Actin (Beyotime) was employed as an internal control. After washing, the membranes were incubated with horseradish peroxidase-conjugated goat anti-rabbit or anti-mouse IgG (Beyotime) and were visualized with an enhanced chemiluminescence detection reagent from Pierce.

## 3. Subcutaneous Implanted Models

The tumor formation assay was performed in male BALB/c nude mice. The animal care and experimental protocols were approved by the Model Animal Research Center of Drum-Tower Hospital and carried out strictly according to Institutional Animal Care and Use guidelines. 1 × 10^7^ Hep-G2 cells (NC and sh-LOXL1-AS1) were subcutaneously injected into the right axilla of 6 BALB/C nude mice. Tumor weights and volumes were measured at the beginning of day 4 after the tumor cell injection. Each mouse was sacrificed, and the tumors were dissected and weighed four weeks later.

### 3.1. Statistical Analysis

Statistical analysis was performed using SPSS 18.0 software. The data were expressed as the mean ± SD. Two-tailed Student's *t*-test was used to determine statistical significance. *P* value of less than 0.05 was considered to be statistically significant.

## 4. Results

**Figure 1:**
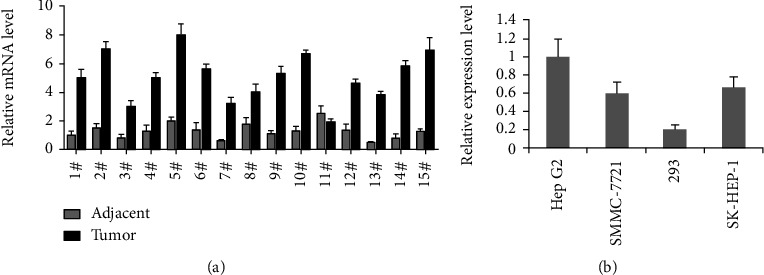
(a) 15 pairs of hepatocarcinoma specimens were detected by qRT-PCR, and the expression level of LOXL1-AS1 in tumor tissue was significantly higher than that in adjacent nontumor tissues. (b) LOXL1-AS1 expressions in three hepatocarcinoma and HEK-293 cell lines were tested by qRT-PCR analysis. The Hep-G2 cell had the highest expression in all four cell lines.

LOXL1-AS1 was overexpressed in hepatocarcinoma tissues and cell lines. We collected cancer and adjacent normal tissues from 15 hepatocarcinoma patients at Affiliated Xinghua People's Hospital of Yangzhou University and assessed the expression level of LOXL1-AS1 RNA in these samples by qRT-PCR. As revealed in Figure 1(a), the expression level of LOXL1-AS1 RNA in tumor tissue was significantly higher than that in adjacent normal tissues (with a median increase of over 3-fold). Besides, 14 out of 15 tumor tissues (93.3%) had a 2-fold or higher expression of LOXL1-AS1 RNA than that in adjacent normal tissues (*P* < 0.05). Cell culture experiments in vitro were used to elaborate molecular mechanism of LOXL1-AS1 in promoting liver cancer development. The LOXL1-AS1 RNA expressions in Hep-G2, SMMC7721, SK-HEP1, and HEK-293 cell lines were tested by qRT-PCR. The HEK-293 cell line was used as the normal control. As revealed in Figure 1(b), the LOXL1-AS1 RNA expression was highest in Hep-G2 cell lines. Because Hep-G2 was reckoned to be a highly invasive and metastatic hepatoma cell line, it was chosen in the following experiments. The RNAi technology was employed to specifically knockdown the endogenous LOXL1-AS1 in Hep-G2 cell line.

### 4.1. Lentiviral shRNA Specifically and Effectively Inhibited LOXL1-AS1 RNA Expression in Hep-G2 Cells

**Figure 2:**
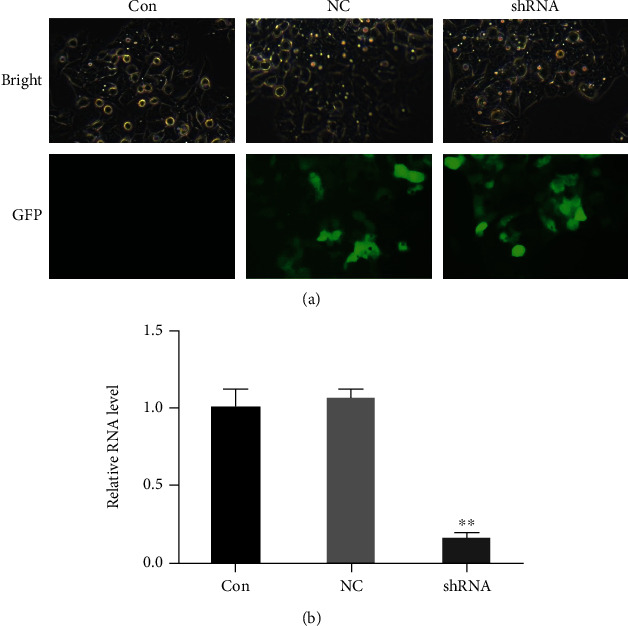
(a) The lentivirus containing LOXL1-AS1 shRNA or control shRNA infected the Hep-G2 cell line. Expression of GFP indicates the lentivirus efficiency of infection in Hep-G2 cells (original magnification ×20). (b) qRT-PCR manifested knockdown efficiency of LOXL1-AS1 in the Hep-G2 cell line. The expression of *β*-actin was used as an internal control. Graph revealed mean ± SD. ^∗∗^ meant *P* < 0.01.

In order to study the loss of LOXL1-AS1 function in the hepatocarcinoma Hep-G2 cell line, a short hairpin RNA (shRNA) which targeted the LOXL1-AS1 gene was inserted into a lentivirus vector so as to inhibit LOXL1-AS1 expression. As verified by the GFP expression 4 days after infection (Figure 2(a)), the proportion of positive cells infected with lentiviruses expressing LOXL1-AS1 shRNA was more than 90%. The qRT-PCR verified that the expression level of LOXL1-AS1 in the Hep-G2 cell line was significantly suppressed by LOXL1-AS1-specific shRNA expression (Figure 2(b)). This indicated that LOXL1-AS1 expression could be effectively downregulated by shRNA in the Hep-G2 cell line.

### 4.2. Suppression of Cell Proliferation in Liver Cancer Cell Line by LOXL1-AS1 Knockdown

**Figure 3:**
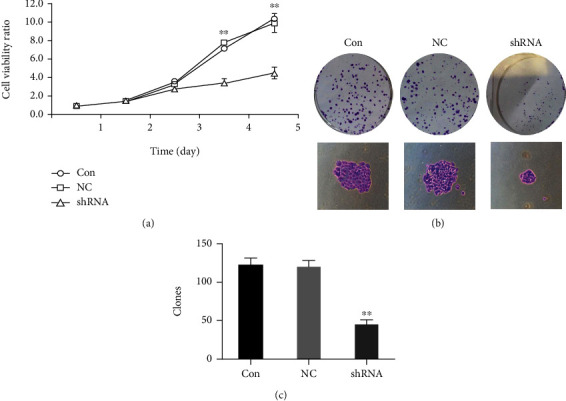
(a) MTT assay manifested that Hep-G2 cell proliferation was inhibited by LOXL1-AS1-specific shRNA expression. The value was expressed as the mean ± SD of three independent experiments (^∗∗^ meant *P* < 0.01). (b, c) Hep-G2 cells infected with Lv-shLOXL1-AS1 or Lv-NC were seeded in 6-well plates and measured 14 days after culturing. (b) Representative Giemsa staining pictures of the colonies by Hep-G2 cells 14 days after plating. (c) The number of Hep-G2 cell colonies after seeding for 14 days was counted (graph revealed mean ± SD, ^∗∗^ meant *P* < 0.01).

To investigate the correlation between LOXL1-AS1 knockdown and proliferation of liver cancer cells in vitro, MTT and colony formation assays were performed.  Figure 3(a) revealed that the proliferation ability of the Hep-G2 cell reduced significantly after infection with sh-LOXL1-AS1 for 5 d in MTT assay (*P* < 0.01). In addition, the colony formation assay also revealed that silencing of LOXL1-AS1 significantly decreased the number and size of Hep-G2 cell colonies (Figures 3(b) and 3(c)) compared with the Con and NC groups (*P* < 0.01).

### 4.3. Inhibition of Cell Migration in Liver Cancer Cell Line by LOXL1-AS1 Knockdown

**Figure 4:**
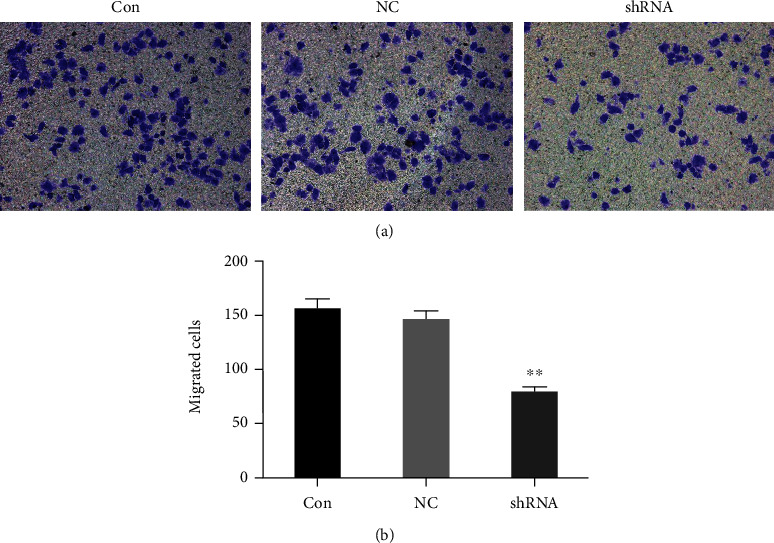
LOXL1-AS1 knockdown suppressed migration in the liver cancer cell line. (a) Representative pictures of crystal violet-stained Hep-G2 cells migrated through the polycarbonate membranes. (b) Statistical plot of the average number of migrated Hep-G2 cells in all three groups. Graph revealed mean ± SD, ^∗∗^ meant *P* < 0.01.

**Figure 5:**
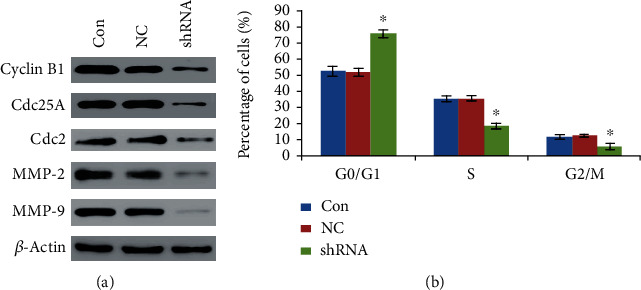
(a) Inhibition of LOXL1-AS1 expression reduced expression of some cell cycle protein and tumor metastasis-associated protein. Western blot analysis revealed the significantly reduced expression levels of Cdc2, Cdc25A, cyclin B1, MMP-2, and MMP-9 in the si-LOXL1-AS1 group compared with the NC and Con groups. (b) Effect of LOXL1-AS1 knockdown on cell cycle progression. The knockdown of LOXL1-AS1 resulted in a significant increase in the percentage of cells in the G0/G1 phase. Graph revealed mean ± SD, ^∗^ meant *P* < 0.05.

In order to evaluate the migrated potential of LOXL1-AS1 to liver cancer cell, we employed Matrigel Transwell Assay to confirm the question in Hep-G2 cells. As revealed in Figures 4(a) and 4(b), the number of migrated cells was significantly decreased in the LOXL1-AS1 knockdown group compared with the control group (*P* < 0.01). Moreover, western blot analysis was employed to determine the effects of LOXL1-AS1 knockdown on the expression of tumor metastasis-associated protein. The expression of matrix metalloproteinases (MMPs) such as MMP-2 and MMP-9 proteins was significantly inhibited in the sh-LOXL1-AS1 group compared with the NC and Con groups (Figure 5(a)). Taken together, the transwell assay indicated that knockdown of LOXL1-AS1 by RNAi technology significantly alleviated hepatocarcinoma cell migration, which might be an important contributor in hepatocarcinoma metastasis.

### 4.4. Inhibition of LOXL1-AS1 Expression Induced G0/G1 Phase Cell Cycle Arrest by Regulating the Expression of Cell Cycle Proteins

We used flow cytometry to validate the action of LOXL1-AS1 silencing on cell cycle progression. For the Hep-G2 cell line, the knockdown of LOXL1-AS1 led to a significant increase in the percentage of cells in the G0/G1 phase (Figure 5(b), *P* < 0.05). This result manifested that knockdown of LOXL1-AS1 inhibited Hep-G2 cell proliferation probably via inducing G0/G1 phase arrest. Moreover, western blot analysis was employed to determine the effects of LOXL1-AS1 knockdown on the expression of cell cycle regulatory protein. The expression of Cdc2, Cdc25A, and cyclin B1 proteins was significantly inhibited in the sh-LOXL1-AS1 group compared with the NC and Con groups (Figure 5(a)). These results implied that depletion of LOXL1-AS1 gave rise to the G0/G1 phase cell cycle arrest, possibly via reducing expression of these cell cycle regulators.

### 4.5. LOXL1-AS1 Knockdown Inhibited Hepatocacinoma Growth In Vivo

**Figure 6:**
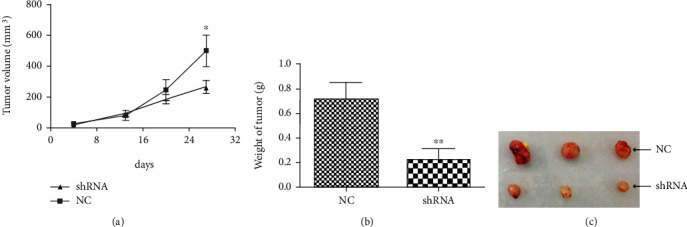
LOXL1-AS1 knockdown inhibited hepatocarcinoma growth in vivo. (a) Tumor growth curve. Hep-G2 cells were stably transfected with sh-LOXL1-AS1 or sh-control and then injected into nude mice (*n* = 3), respectively. Tumor volume was calculated from day 4 after injecting tumor cells. The error bars indicated the standard deviation (SD). (b) Tumor weights were expressed as mean ± S.D when the tumors were removed from the nude mice. (c) The number and size of tumors after removing from the mice. Graph revealed mean ± SD, ^∗^ meant *P* < 0.05, ^∗∗^ meant *P* < 0.01.

To further explore the effect of LOXL1-AS1 knockdown on cell tumorigenesis in vivo, Hep-G2 cells stably transfected with sh-LOXL1-AS1 or sh-control were inoculated into male nude mice. Four weeks after injection, compared with the control group, LOXL1-AS1 knockdown dramatically inhibited tumor growth as demonstrated by reduced tumor weight and size (Figure 6). Together, silencing of LOXL1-AS1 might reduce the growth of established hepatocarcinoma xenografts.

## 5. Discussion

Although diagnosis and treatment modalities such as surgical resection, transplantation, ablation, radiotherapy, chemotherapy, and targeted drug sorafenib have achieved great progress, HCC has been still a great challenge for physicians and patients [18]. The poor prognosis of HCC is mainly due to the strong recurrence and metastasis. Therefore, understanding the molecular mechanism and key regulators in the process of metastasis is very important for medical workers to improve curative effect and predict prognosis.

It is increasingly apparent that mammalian genomes encode thousands of lncRNAs regulating gene expression. Most previous researches [19–21] about long noncoding RNA LOXL1 have always focused on exfoliation syndrome or glaucoma. LOXL1-AS1 is a long noncoding RNA encoding the opposite strand of LOXL1. Hauser et al. reported that LOXL1-AS1 expression was significantly altered in response to oxidative stress and the findings implied dysregulation of its expression by genetic risk variants played a key role in exfoliation syndrome pathogenesis [17]. However, up to now, little is known about the role of LOXL1-AS1 in tumorigenesis and development. Recently, LOXL1-AS1 has been reported to regulate tumor occurrence, progression, and growth in several cancers. Dong et al. reported that LOXL1-AS1 may promote invasion and metastasis via inhibiting miR-708-5p expression and activity in breast cancer [22]. Another research demonstrated that lncRNA LOXL1-AS1 affected ovarian cancer cell growth, migratory, and invasion by modulating the miR-18b-5p/VMA21 axis [23]. It was reported that LOXL1-AS1 had an influence on the proliferation and migration of several other cancers by regulating some key genes [24, 25]. In the present study, we observed that the expression level of LOXL1-AS1 in liver cancer tissue was significantly higher than that in adjacent normal tissues and its expression in three hepatic carcinoma cell lines was obviously higher than that in a normal cell line. This indicated that the abnormal expression of LOXL1-AS1 might be associated with the biological behavior of HCC.

During recent years, more and more researches have focused on the function of lncRNA in tumor growth, invasion, and metastasis [26–28]. Therefore, identifying and investigating tumor-associated lncRNAs may supply novel prognostic biomarkers or therapeutic strategies to tumor. lncRNA HULC was the first reported to be specifically upregulated in HCC [29]. Other lncRNAs, such as HOTTIP/HOXA13 [30], PCNA-AS1 [31], and TUC338 [32], have been described to be related to the development and progression of HCC. To investigate LOXL1-AS1 biological function in HCC, a lentivirus-mediated RNAi technology was employed to suppress its expression. We discovered that, in Hep-G2 cell line, LOXL1-AS1 downregulation significantly inhibited cell proliferation in the light of the MTT and colony formation assays in vitro. In addition, animal experiment in vivo was consistent with the findings. What is more, cell migration was also inhibited in vitro in Matrigel Transwell Assay by LOXL1-AS1 knockdown. Based on these results, we inferred that lncRNA LOXL1-AS1 might play an important role in the occurrence and development of HCC and that it might be a significant prognostic factor and therapeutic target in HCC.

To further explore the possible biological mechanisms of LOXL1-AS1 in HCC pathogenesis, flow cytometry was used to validate the effect of LOXL1-AS1 silencing on cell cycle progression. Obviously, cell cycle progression relies on the activation of cyclin-dependent kinases (CDKs) and their related regulatory subunits, the cyclins [33]. In the present study, cell cycle analysis revealed that knockdown of LOXL1-AS1 induced significantly a G0/G1 phase cell cycle arrest, which might be partly attributed to the downregulation of Cdc2, Cdc25A, and cyclin B1 protein expression. Thus, LOXL1-AS1 may regulate the expression level of cell cycle regulatory factors. However, the precise molecular mechanism by LOXL1-AS1 knockdown induces cell cycle arrest is still needed to further study. Moreover, we employed western blot to determine the effects of LOXL1-AS1 knockdown on the expression of tumor metastasis-associated proteins. Our results implied that the knockdown of LOXL1-AS1 significantly alleviated hepatocarcinoma cell migration, which might be partly attributed to the reduction of MMP-2 and MMP-9 protein expressions.

Together, we discovered that LOXL1-AS1 was significantly upregulated in HCC tissues. Knockdown of LOXL1-AS1 could inhibit HCC cell proliferation, migration, and invasion in vitro and reduce its growth in vivo. Therefore, LOXL1-AS1 might serve as an important promoter in liver cancer proliferation and migration and potential therapeutic target for HCC.

## Figures and Tables

**Table 1:** The clinicopathological characteristics in hepatocarcinoma patients.

Parameters	Case
Age (years)	
<55	6
>55	9
Gender	
Male	8
Female	7
TNM stage	
I-II	5
III-IV	10
Lymphatic metastasis	
Yes	11
No	4

## Data Availability

All data and code generated or used during the study appear in the submitted article.
