# Expression of Concern on “The Long Noncoding RNA LOXL1-AS1 Promotes the Proliferation, Migration, and Invasion in Hepatocellular Carcinoma”

**DOI:** 10.1155/2022/9867912

**Published:** 2022-04-27

**Authors:** Analytical Cellular Pathology

*Analytical Cellular Pathology* would like to express concern with the article titled “The Long Noncoding RNA LOXL1-AS1 Promotes the Proliferation, Migration, and Invasion in Hepatocellular Carcinoma” [1] because it is unclear where this work was conducted and whether the necessary ethical approvals were in place. The article states that ethics approval was granted by Nanjing Medical University, whilst the authors are shown to be affiliated with Xinghua Hospital affiliated to Yangzhou, where the patient data appears to have been obtained. The authors were contacted for clarifications as to the discrepancy but have been unresponsive.
